# Combining rare and common genetic variants improves population risk stratification for breast cancer

**DOI:** 10.1016/j.gimo.2024.101826

**Published:** 2024-02-02

**Authors:** Alexandre Bolze, Daniel Kiser, Kelly M. Schiabor Barrett, Gai Elhanan, Jamie M. Schnell Blitstein, Iva Neveux, Shaun Dabe, Harry Reed, Alexa Anderson, William J. Metcalf, Ekaterina Orlova, Ildiko Thibodeau, Natalie Telis, Ruomu Jiang, Nicole L. Washington, Matthew J. Ferber, Catherine Hajek, Elizabeth T. Cirulli, Joseph J. Grzymski

**Affiliations:** 1 Helix, San Mateo, CA; 2 Department of Internal Medicine, University of Nevada Reno, School of Medicine, Reno, NV; 3 Renown Health, Reno, NV; 4 Department of Human Genetics, University of Pittsburgh, Pittsburgh, PA

**Keywords:** *ATM* and *CHEK2*, Breast cancer, Family history, Polygenic risk score, Population screening

## Abstract

**Purpose:**

This study aimed to evaluate the performance of different genetic screening approaches to identify women at high risk of breast cancer in the general population.

**Methods:**

We retrospectively studied 25,591 women with available electronic health records and genetic data, participants in the Healthy Nevada Project.

**Results:**

Family history of breast cancer was ascertained on or after the record of breast cancer for 78% of women with both, indicating that this risk assessment method is not being properly utilized for early screening. Genetics offered an alternative method for risk assessment. 11.4% of women were identified as high risk based on possessing a predicted loss-of-function (pLOF) variant in *BRCA1*, *BRCA2*, or *PALB2* (hazard ratio = 10.4, 95% confidence interval: 8.1-13.5) or a pLOF variant in *ATM* or *CHEK2* (hazard ratio = 3.4, CI: 2.4-4.8) or being in the top 10% of the polygenic risk score (PRS) distribution (hazard ratio = 2.4, CI: 2.0-2.8). Moreover, women with a pLOF in *ATM* or *CHEK2* and ranking in the top 50% of the PRS displayed a high risk (39.2% probability of breast cancer at age 70), whereas their counterparts in the bottom 50% of the PRS were not at high risk (14.4% probability at age 70).

**Conclusion:**

Our findings suggest that a combined monogenic and polygenic approach allowed a better identification of participants with high risk while minimizing false positives.

## Introduction

Disparities pervade all aspects of breast cancer screening and treatment. Current clinical risk assessments, aimed at identifying women eligible for referral to high-risk breast cancer clinics, are often inconsistently applied, leading to inequitable outcomes.^1^, ^2^, ^3^ In 2021, Nevada attempted to address this issue by enacting a law mandating payors to cover counseling and genetic testing for women identified as high-risk for breast cancer. However, consistent implementation of screening and precise capture of family history data by all providers remains a crucial first step in assessing who should receive counseling and genetic testing.

An alternative approach to identifying individuals who may benefit from referral to a high-risk clinic is to adopt a population-level genetic screening approach, sequencing all women.^4^, ^5^, ^6^, ^7^ The detection of rare, high-impact variants, such as loss-of-function variants in *BRCA1* and *BRCA2*, is used to identify individuals who should undergo mammography screening at an earlier age and with greater frequency,^4^^,^^8^ have breast MRIs, and should consider risk-reducing measures. Variants in other genes, including *PALB2*, *ATM*, and *CHEK2*, are also strongly associated with breast cancer risk.^9^, ^10^, ^11^, ^12^ However, a significant association with breast cancer may not warrant screening the entire population, especially if the positive predictive value in the general population is low.^13^ Additionally, polygenic risk scores (PRS), based on common variants associated with breast cancer, have been developed and replicated across numerous cohorts.^14^^,^^15^ In the United Kingdom, a score based on 313 SNVs has been integrated in BOADICEA to create a comprehensive breast cancer risk prediction model,^16^ which aims to identify individuals with high or low risk for breast cancer. Despite the apparent benefits of using genetic information to identify women at high risk of breast cancer, particularly women with incomplete family history, many questions remain about the real-world impact of such a measure and the most equitable way to implement it across the population in the United States.

The Healthy Nevada Project (HNP) is an all-comer genetic screening and research project based in northern Nevada. HNP participants consent to (1) provide a sample for clinical exome sequencing and sequencing of 100,000s of common SNVs outside of the exome, (2) receive notifications of CDC Tier 1 findings, and (3) participate in research. Currently, a report of clinically relevant findings is returned for 3 CDC Tier 1 genomic conditions: hereditary breast and ovarian cancer (genes: *BRCA1* and *BRCA2*), Lynch syndrome (genes: *MLH1*, *MSH2*, *MSH6*, and *PMS2*), and familial hypercholesterolemia (genes: *APOB*, *LDLR*, and *PCSK9*). These conditions were defined by CDC’s Office of Public Health Genomics as those having significant potential for positive impact on public health. Research is enabled by linking of sequencing data with available electronic health records (EHR), regular refresh of the EHR, and recontact for health surveys and participation in future studies. This study, therefore, offers opportunities to retrospectively evaluate various genomic screening methods for identifying women at high risk of breast cancer and to prospectively assess the impact and outcomes of these methods when applied in a clinical setting.

The objectives of this study are to (1) evaluate the effectiveness of family history as a tool for identifying women at high risk of breast cancer, (2) determine the impact of pathogenic variants in *BRCA1*, *BRCA2*, *PALB2*, *ATM*, and *CHEK2*, as well as the presence of a high polygenic risk, on breast cancer diagnosis within the HNP cohort, and (3) characterize the population of women newly identified or missed based on the utilization of different combinations of genetic and family history information. Many studies and institutions have their own definitions and thresholds for what qualifies as “high risk” for breast cancer.^16^, ^17^, ^18^ In this study, we define “high risk” of breast cancer as a 20% or greater risk of breast cancer by age 70, corresponding to >2× the average risk for women in the United States.

## Materials and Methods

### Subjects

This study was based on the HNP. The HNP study was reviewed and approved by the University of Nevada, Reno Institutional Review Board (IRB, project 956068-12), and all participants provided informed consent. The initial data set comprised 47,179 individuals. For this study, we only included participants who were inferred to be of female sex based on the genetic data and had longitudinal data length >0 in the EHR at Renown Health.

### Clinical phenotypes from EHR

Phenotypes were processed from Epic/Clarity Electronic Health Records (EHR) data. For this study, we focused on breast cancer and did not look at ovarian cancer despite the known impact of pathogenic variants in *BRCA1* and *BRCA2* on both breast and ovarian cancers. The mean number of ICD10-CM codes recorded per participant was 90 (median: 67). ICD10-CM codes C50, D05, and Z85.3 were used to identify women diagnosed with breast cancer (Supplemental Figure 1). We did not take into account secondary neoplasm of the breast.

To test whether there may be a general recording bias in the EHR, and more generally to QC the EHR data, we looked at a few other phenotypes: type 1 diabetes, type 2 diabetes, hypertension, and rheumatoid arthritis. Phenotypes were selected based on the following criteria: (1) not cancers, (2) are common in the population (ideally rates similar to breast cancer), and (3) have a similar age of onset or first diagnosis as breast cancer. We used the following ICD10-CM codes: codes starting with E10 for type 1 diabetes, codes starting with E11 for type 2 diabetes, codes starting with I10 for hypertension, and codes starting with M06 for rheumatoid arthritis.

### Family history information

We compared multiple sources of information to identify individuals with family history. We decided to utilize diagnosis codes (ICD-10 codes) for our main analysis based on our comparisons and the fact that diagnosis codes will make it easier for others to reproduce our analysis. For family history of breast cancer, we used ICD10-CM code Z80.3. For family history of other conditions, we first excluded all Z80.3 codes, and then included all codes starting with Z80, Z81, Z82, Z83, or Z84.

In summary, 3 sources of family history records for breast cancer were identified in the EHR: diagnosis codes, a family history table (FHx table), and a table containing responses to the 7-question family history screening (FHS7 table). Using data from August 2021, we evaluated the agreement between these 3 sources for identifying patients with a family history of breast cancer (Supplemental Figure 2A). The family history table source was the most comprehensive followed by diagnoses codes.

We further evaluated whether using the family history table in addition to diagnosis codes would meaningfully change our conclusions about the effectiveness of family history for evaluating risk of breast cancer. To do this, we examined the temporal association of family history documentation with a diagnosis of breast cancer in 2 ways: determining dates of family history of breast cancer using (1) diagnosis codes only or using (2) both diagnosis codes and FHx table. The difference in temporal association when FHx table is included or excluded appears to be negligible (Supplemental Figure 2B), despite the overall increase of participants with a positive family history. As a further test, we examined the temporal association between entries in FHx table and breast cancer without including diagnosis codes to see if entries in FHx table also tended to appear after breast cancer diagnosis had already been diagnosed. We found that among 371 patients with both breast cancer and a breast cancer entry in the FHx table, only 42.3% (157 of 371) had their 1st breast cancer entry in FHx table before their diagnosis of breast cancer (Supplemental Figure 2C). 30.4% (65 of 214) of patients with their 1st breast cancer entry in the FHx table after or at the same time as their breast cancer diagnosis had a family entry for another condition before the breast cancer diagnosis. These results validated the results reported in the main text (using Family history diagnosis codes only) showing that family history of breast cancer often fails to be documented before breast cancer, even if family history was previously assessed in some manner. Based on these analyses, we concluded that diagnosis codes were sufficient for our purposes in evaluating the effectiveness of family history for breast cancer risk screening.

### Sequencing

The samples were sequenced at Helix using the Exome+ assay, which includes a clinical exome and the sequencing of SNVs outside of the exome. Data were processed using a custom version of Sentieon and aligned to GRCh38, with variant calling and phasing algorithms following Genome Analysis Toolkit best practices. Imputation of common variants in the HNP data was performed by pre-phasing samples and then imputing. Pre-phasing was performed using reference databases, which include the 1000 Genomes Phase 2 data. This was followed by genotype imputation for all 1000 Genomes Phase 3 sites that have genotype quality values <20.

### List of genes mentioned in the study

*BRCA1*, HGNC ID: 1100

*BRCA2*, HGNC ID: 1101

*PALB2*, HGNC ID: 26144

*ATM*, HGNC ID: 795

*CHEK2*, HGNC ID: 16627

*BARD1*, HGNC ID: 952

*RAD51C*, HGNC ID: 9820

*RAD51D*, HGNC ID: 9823

*TP53*, HGNC ID: 11998

*CDH1*, HGNC ID: 1748

*PTEN*, HGNC ID: 9588

*STK11*, HGNC ID: 11389

*MLH1*, HGNC ID: 7127

*MSH2*, HGNC ID: 7325

*MSH6*, HGNC ID: 7329

*PMS2*, HGNC ID: 9122

*APOB*, HGNC ID: 603

*LDLR*, HGNC ID: 6547

*PCSK9*, HGNC ID: 20001

### Variant annotation and classification

We focused on identifying variants in 5 genes: *BRCA1*, *BRCA2*, *PALB2*, *ATM*, and *CHEK2*. Although some studies only look at the common del1100C variant in *CHEK2*, we decided to analyze all *CHEK2* predicted loss-of-function (pLOF) variants. Other genes are known to be associated with risk of breast cancer. *BARD1*, *RAD51C*, and *RAD51D* have been included in the BOADICEA algorithm in 2022, and rare variants in *TP53*, *CDH1*, *PTEN*, and *STK11* have high-penetrance for breast cancer. However, we decided to not include these genes because these genes did not come up when looking at an unselected population. It would therefore be very difficult to provide good estimates for the risk conferred by these genes in our study, let alone being able to study the combination of rare variants in these genes with polygenic risk.^19^^,^^20^

Our strategy to identify variants increasing the risk of breast cancer (sometimes labeled as pathogenic or likely pathogenic) was based on 2 philosophical directions: (1) our study is focused on population-based screening and that many of the ACMG criteria that would apply for a diagnostic test do not apply or cannot be calculated in the context of screening a healthy individual,^21^ and (2) this is a research study, and an automated variant annotation is more reproducible than a manual interpretation while still providing very similar results. Notably, our approach was similar to the approach taken in the Geisinger’s MyCode research project, which pioneered population-scale proactive genomic screening.^22^

The following steps were done to annotate variants and identify loss-of-function variants (Supplemental Figure 3A):

1. Preparation of the genetic file. Genotype processing was performed in Hail version 0.2.115-10932c754edb (https://github.com/hail-is/hail/commit/10932c754edb). Restrict Hail matrix table to specific genomic intervals for the 5 genes.
2. Annotate with Ensembl Variant Effect Predictor-99,^23^ with Clinvar (file clinvar_20220723.vcf.gz downloaded from https://ftp.ncbi.nlm.nih.gov/pub/clinvar/vcf_GRCh38/ was used) and with gnomAD_v3 (https://gnomad.broadinstitute.org/downloads#v3-variants). The MANE transcripts were used to determine variant consequence.^24^ There were 13,853 variants in the 5 genes before any filtering.
3. Filter out variants flagged as “Filtered variants” by gnomAD because they did not pass their quality control process. 13,673 variants remained.
4. We then did the annotation using the following logic:
  a. IF the variant was rs555607708 *CHEK2* del1100C variant (ENST00000404276.6:c.1100del) THEN label as “P/LP.”
  b. ELSE IF the variant was reviewed by the Clingen expert panel (CLNREVSTAT field in Clinvar table) THEN keep the interpretation from the Clingen expert panel.
  c. ELSE IF the variant had criteria provided by multiple submitters with no conflicts (CLNREVSTAT field in Clinvar table) and was “Benign” or “Likely Benign” (CLNSIG field) THEN label as “B/LB.”
  d. ELSE IF the variant had criteria provided by multiple submitters with no conflicts (CLNREVSTAT field in Clinvar table) and was “Uncertain significance” (CLNSIG field) THEN label as “VUS.”
  e. ELSE IF the variant was called as LOF with High Confidence by LOFTEE,^25^ THEN label “P/LP.” Briefly, LOFTEE flags LOF variants as low confidence (LC) if the LoF version is the ancestral state, if they are a stop gain or frameshift near the end of the gene or are in an exon with non-canonical splice sites around it, or if they are a splice variant that is not predicted to affect the splicing of a coding exon.
  f. ELSE label variant as “not pathogenic.”
5. A total of 184 variants were called “P/LP” this way.
6. Additional quality control was done for each of these variants, including review of the DP, AD, GQ fields, the allele frequency in our cohort and in other databases, and visualization of the BAM file with IGV for small insertions or deletions.
7. Two variants in the same gene and same individual were removed as likely false positives after reviewing the BAM file. A total of 182 variants were annotated as pathogenic variants for this study.

Notably, this strategy utilizes databases such as Clinvar, which have some biases. For example, there are more VUSs for variants more frequent in populations of non-European genetic similarity.

### CNV annotations

The Helix Exome+ assay includes a copy-number variant (CNV) caller, allowing us to incorporate rare CNVs at exon-level resolution. Briefly, CNVs with the PASS QC filter were annotated with overlapping MANE transcripts. Only large deletions were considered to be pathogenic for this study.

### PRS calculation

The PRS model selected is the 313 SNVs PRS published in 2019.^14^ It is also available in the PGS catalog^26^ (https://www.pgscatalog.org/score/PGS000004/). We first converted the coordinates and effect size of each alternate allele from human reference genome GRCh37 to the more recent GRCh38. We used 300 SNVs out of the 313 to ensure we had strong overall callability for each SNV used and confidence that the alternate (and effect) allele was correct. Allele frequencies of alternative alleles matched closely with published allele frequencies for these variants. (Supplemental Figure 4B).

We then calculated the score in each of the 25,591 women included in the study. The distribution of genetic similarity was the following: N Africa = 499, N Americas = 3,728, N East Asia = 832, N Europe = 19,484, N Other = 929, and N South Asia = 119. Briefly, a genotype dosage was calculated for each variant in the score for each individual. The dosage was based on the genotype probability field resulting from the imputation pipeline. When an individual had no GP or no GT (genotype) for a specific variant, the dosage was based on the allele frequency of this variant in gnomAD v3 for the population closest to the genetic similarity of the participant. We then split the cohort into 6 cohorts based on genetic similarity and ranked individuals based on their PRS value and assigned a percentile based on the ranking within the participant’s genetic similarity distribution.

Lastly, we regrouped all 6 genetic similarity groups into 1 cohort for later analyses that were based on percentiles.

### Cancer risk thresholds

Different definitions and thresholds exist to stratify women considered at high risk of breast cancer.

• The National Comprehensive Cancer Network (NCCN) guidelines consider “Increased risk” an asymptomatic women with a residual lifetime risk ≥20% as defined by models that are largely based on family history.^18^
• The American Cancer Society considers women at high risk as those who have a lifetime risk of breast cancer of about 20% to 25% or greater, according to risk assessment tools that are based mainly on family history.^17^
• The UK NICE (National Institutes of Clinical and Healthcare Excellence) guidelines use a threshold of >30% at age 80 for “high risk” and between 17 and 30% at age 80 for “moderate risk.”^16^

Here, we followed the NCCN definition and considered a woman to be at increased risk or high risk if they had an accumulated risk of being diagnosed with breast cancer ≥20% by age 70.

### Survival analysis, hazard ratios, and statistical tests

The specific tests used are described in detail in the main text or in the figure legends.

Kaplan Meier survival curves were done using the KaplanMeierFitter function from the Lifelines python library.

Statistical differences between survival curves were assessed using a logrank_test function from the lifelines.statistics python library.

Values at a given age (eg, 70 years old) were calculated using the “predict” function.

Hazard ratios were calculated using the CoxPHFitter function from the Lifelines python library.

Plots were made using pyplot from the matplotlib python library.

### Genetic screening evaluation

• Positive Predictive Value (PPV) was calculated as: (% of Positives that were diagnosed with breast cancer by age 70 based on the KM curve).

PPV_strat1 = 34%

PPV_strat2 = 23%

• Specificity = TN/(TN + FP).

Here, true negatives (TN) are defined as those who were put in the “average risk group” based on genetic screening and with no breast cancer diagnosis by age 70.

False positives (FP) are defined as those who were put in the “high risk group” and who did not have a breast cancer diagnosis by age 70.

Specificity_strat1 = 23,593 × (1 − 0.076)/(23,593 × (1 − 0.076) + 1998 × 0.66) = 21,800/(21,800 + 1319) = 21,800/23,119 = 94.3%

Specificity_strat2 = 22,682 × (1 − 0.081)/(22,682 × (1 − 0.081) + 2909 × 0.77) = 20,845/(20,845 + 2240) = 20,845/23,085 = 90.3%

• Number Needed to Screen (NNS): The number of women who must be enrolled in our population genetic screening program to prevent 1 adverse outcome. Here, we defined preventable adverse outcome a diagnosis of breast cancer before age 50.

NNS_strat1 = 1/(fraction with genetic high risk × fraction of those with a breast cancer diagnosis by age 50)

NNS_strat1 = 1/(1998/25,591 × 0.070) = 1/(0.078 × 0.070) = 1/0.00547 = 183.

NNS_strat2 = 1/(2909/25,591 × 0.041) = 1/(0.114 × 0.041) = 1/0.0047 = 213.

## Results

### Demographics of the HNP

As of December 2022, 47,179 individuals had consented to and been sequenced as part of the HNP. Efforts were made to increase the diversity of participants in the HNP and to ensure that these participants were representative of the overall patient population of Renown Health. Despite these efforts, there was a higher proportion of participants who self-reported their race to be White (83.7% in HNP compared with 76.8% in Renown Health) (Supplemental Table 1).^4^^,^^27^ Our analysis focused on women with available EHR from Renown Health. In total, we analyzed the genetic and clinical information of 25,591 participants (Table 1). The mean age was 53.8 years, with a bimodal age distribution (Supplemental Figure 1A). Of these participants, 4977 (19.4%) were 70 years or older in 2022. We utilized EHR diagnosis tables to identify participants diagnosed with breast cancer. In total, 1295 women (5.1%) had at least 1 ICD10-CM code starting with C50 (indicating malignant neoplasm of the breast), D05 (indicating carcinoma in situ of the breast), or Z85.3 (indicating personal history of malignant neoplasm of the breast) (Table 1, Supplemental Figure 1B and C). Among those 70 years of age or older in 2022, 570 (11.5%) had a diagnosis of breast cancer. This prevalence is consistent with estimates from the National Cancer Institute, reporting that 12.9% of women born in the United States today will develop breast cancer at some time during their lives.^28^

**Table 1:** Demographics of participants from the Healthy Nevada Project included in this study

Demographic Characteristic	All Participants in the Study	Participants ≥70 Years Old in 2022
*N* female participants	25,591	4977
Age in 2023	Mean: 53.8 (range: 19-89+)	Mean: 77.2 (71-89+)
Genetic similarity group	Africa: 499 (1.9%)	Africa: 33 (0.7%)
	Americas: 3728 (14.6%)	Americas: 255 (5.1%)
	East Asia: 832 (3.3%)	East Asia: 110 (2.2%)
	Europe: 19,484 (76.1%)	Europe: 4501 (90.4%)
	Other: 929 (3.6%)	Other: 78 (1.6%)
	South Asia: 119 (3.6%)	South Asia: 0 (0%)
Length of Electronic Health Record	Mean: 12.0 years	Mean: 13.4 years
Diagnosis of breast cancer^a^	1295 (5.1%)	570 (11.5%)

a Based on ICD10-CM codes starting with: C50, D05, or Z85.3.

### Family history misses most women at increased risk of breast cancer before diagnosis

**Figure 1:**
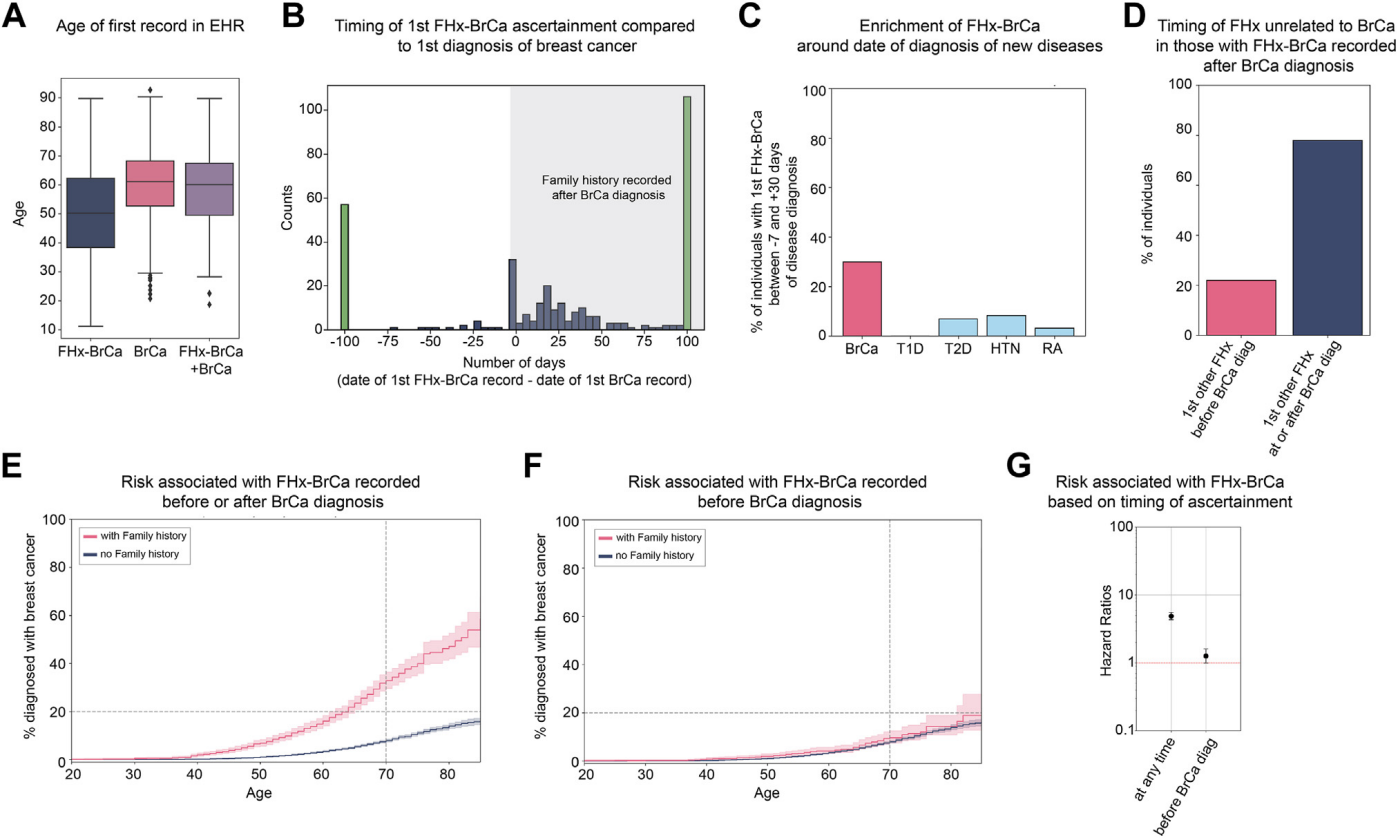
**Impact of family history of breast cancer (FHx-BrCa) on risk of breast cancer (BrCa).** A. Mean age of first record in the electronic health records. *N =* 1772 for FHx-BrCa; *N =* 1295 for BrCa; *N =* 331 for FHx-BrCa and BrCa. B. Days between 1st record of FHx-BrCa and 1st record of BrCa. All bars on the left side (negative numbers) indicate individuals for whom FHx-BrCa was recorded before a BrCa diagnosis. Green bars indicate points before −100 days or after +100 days. C. Percentage of individuals with the 1st record of FHx-BrCa within a time range of −7 to +30 days around the 1st diagnosis of BrCa (pink bar) or other conditions (light blue bars). From left to right: type 1 diabetes (T1D), type 2 diabetes (T2D), hypertension (HTN), and rheumatoid arthritis (RA). D. Timing of 1st family history (FHx) record other than FHx-BrCa for individuals with the 1st FHx-BrCa recorded within 1 week or after the 1st BrCa record. Graph limited to those with at least 1 family history code unrelated to BrCa. *N =* 162. Pink bar: FHx unrelated to BrCa was recorded before BrCa diagnosis and also before FHx-BrCa. Blue bar: FHx unrelated to BrCa was recorded after BrCa diagnosis. E. Kaplan Meier curves showing the % of women with a breast cancer diagnosis by age. Pink curve: with FHx-BrCa, *N =* 1772. Blue curve: without FHx-BrCa, *N =* 23,819. F. Kaplan Meier curves showing the % of women with a breast cancer diagnosis by age. Participants with a 1st FHx-BrCa recorded within 1 week of 1st BrCa diagnosis or after 1st BrCa diagnosis were excluded from analysis. Pink curve: with FHx-BrCa, *N =* 1513. Blue curve: without FHx-BrCa, *N =* 23,819. G. Cox proportional hazard ratios and their 95% Confidence Intervals. *y-*axis is on a log scale.

Current clinical guidelines heavily depend on family history to identify women at increased risk for breast cancer.^29^ We identified 3 sources of breast cancer family history records in the EHR: diagnosis codes, a family history table, and a table containing responses to the 7-question family history screening that was implemented in 2021 to comply with SB251. We evaluated the agreement between these 3 sources for identifying patients with a family history of breast cancer (FHx-BrCa) (Supplemental Figure 2). We used only diagnosis codes to identify women with a FHx-BrCa in subsequent analyses because diagnoses codes data were available for all participants, and diagnoses codes are more standardized and widely used, making it easier for others to reproduce this analysis. However, we recognize the limitations of relying on diagnosis codes of family history rather than more detailed risk factors included in the FHS-7 screening questions. A total of 1772 women (6.9%) had at least 1 ICD10-CM Z80.3 code indicating FHx-BrCa. The mean age of the first code was 50.2 years (Figure 1A). However, the mean age of the first FHx-BrCa code for women diagnosed with breast cancer was 58.6 years, closer to the mean age for breast cancer diagnosis (60.2 years) (Figure 1A), suggesting that family history may be taken at the time of diagnosis. We next examined the specific timing of the family history record and breast cancer diagnosis (Figure 1B). For the majority of participants with both a family history and a diagnosis of breast cancer, the family history ascertainment was recorded at the same time or after the breast cancer diagnosis (Figure 1B). There were 30% of first FHx-BrCa records simultaneous with the first breast cancer diagnosis record (between 1 week before and 1 month after) and 48% occurring afterward (1 month to 15 years after) (Figure 1B).

Two main hypotheses could explain this result: (1) there is a recording bias in the EHR, and many codes are recorded at the same time in the EHR (for example, the first encounter in a new health system), or (2) there is an ascertainment bias, and FHx-BrCa is more often ascertained when there is a suspicion of BrCa. To test the first hypothesis, we looked at the timing of the record of FHx-BrCa in relation to the diagnosis date of another common unrelated disease. There was no enrichment for the FHx-BrCa code to be at the same time as the diagnosis of T2D (χ^2^ test for comparison with enrichment seen between FHx-BrCa and BrCa diagnosis, *P* = 1.9e−11), or other common non-cancer conditions with a similar or earlier average age of onset to breast cancer (Figure 1C). This suggests that the bias observed around breast cancer diagnosis was not a recording bias due to a general effect of receiving more information about a patient at certain time points. We next looked at codes for family history of other conditions. 26.8% of women had at least 1 FHx code not for FHx-BrCa code. Among women with FHx-BrCa recorded simultaneously or after the first BrCa diagnosis, 162 (out of 259) had at least 1 FHx code unrelated to BrCa. Among those, 22% (35 of 162) had at least 1 FHx code for a condition other than BrCa recorded before the BrCa diagnosis and before the FHx-BrCa code (Figure 1D, Supplemental Figure 2C), indicating again that the bias observed was likely due to an ascertainment bias rather than a bias in recording information in the EHR. A third hypothesis could explain why FHx-BrCa was only recorded after the initial BrCa diagnosis or after other FHx codes, which is that FHx-BrCa did not become apparent until later (the family member was not diagnosed with breast cancer yet or patient was unaware of BrCa in relative at time of their diagnosis). We did not specifically test this third hypothesis because, in this case, family history has no value for diagnosing breast cancer earlier, by definition. Overall, these results demonstrate that a patient is more likely to be ascertained for FHx-BrCa on or after a diagnosis of breast cancer or suspicion of breast cancer.

Despite this limitation, we assessed the risk of developing breast cancer for women who were aware of and reported a family history of breast cancer. Among women with a FHx-BrCa code in their medical records, 18.7% (331 of 1772) had a diagnosis of breast cancer, compared with 4.0% (964 of 23,819) of women without, and the hazard ratio (HR) was 4.9 (95% CI: 4.3-5.5, *P* = 1.7e−166, log-rank test) (Figure 1E). To minimize the impact of family history ascertainment bias, we next analyzed the risk associated with a positive family history of breast cancer after removing participants whose first FHx-BrCa code appeared simultaneously with or after the first diagnosis of breast cancer. In this scenario, 4.8% (72 of 1513) of women with a positive family history had a diagnosis of breast cancer, a risk similar to women without a family history (HR = 1.3, CI: 1.0-1.6, *P* = .05) (Figure 1F and G). However, this analysis likely overcorrected the ascertainment bias because it removed the majority of cases among the group with a family history and did not remove cases in the group without a family history. Overall, these results showed that at least 6.9% of women had a positive family history of breast cancer, but family history is often ascertained at or after the time of diagnosis.

### Loss-of-function variants in *PALB2*, *ATM*, and *CHEK2* significantly increase risk of breast cancer

As an alternative to family history, we examined 5 genes—*BRCA1*, *BRCA2*, *PALB2*, *ATM*, and *CHEK2*—in which haploinsufficiency is known to be associated with breast cancer risk based on previous studies doi:10.1101/2022.06.16.22276246.^13^^,^^16^^,^^30^ We thus considered high-confidence pLOF pathogenic variants in these 5 genes and pathogenic variants in ClinVar reviewed by an expert panel. We also examined pathogenic deletions (copy number of 1 or 0) of 1 or more protein-coding exons of the MANE transcript in these genes. Details of variant annotation and classification are in the methods (Supplemental Figure 3A). The variant calls for these genes were of high quality (Supplemental Figure 3B and C), and all variants annotated as pathogenic had an allele frequency below 0.1% in the population, with the exception of the well-known *CHEK2* del1100C variant (ENST00000404276.6:c.1100del) (Supplemental Figure 3D). The list of identified pathogenic single-nucleotide variants or small indels is in Supplemental Table 2, and the list of larger deletions is in Supplemental Table 3. In total, we identified 410 (1.6%) women who had a heterozygous pathogenic variant in 1 of these 5 genes (Supplemental Table 4). No individual had a homozygous pathogenic variant or a copy number of 0 in these genes. One individual had 2 heterozygous pathogenic variants in different genes (1 in *BRCA1* and 1 in *BRCA2*).

**Figure 2:**
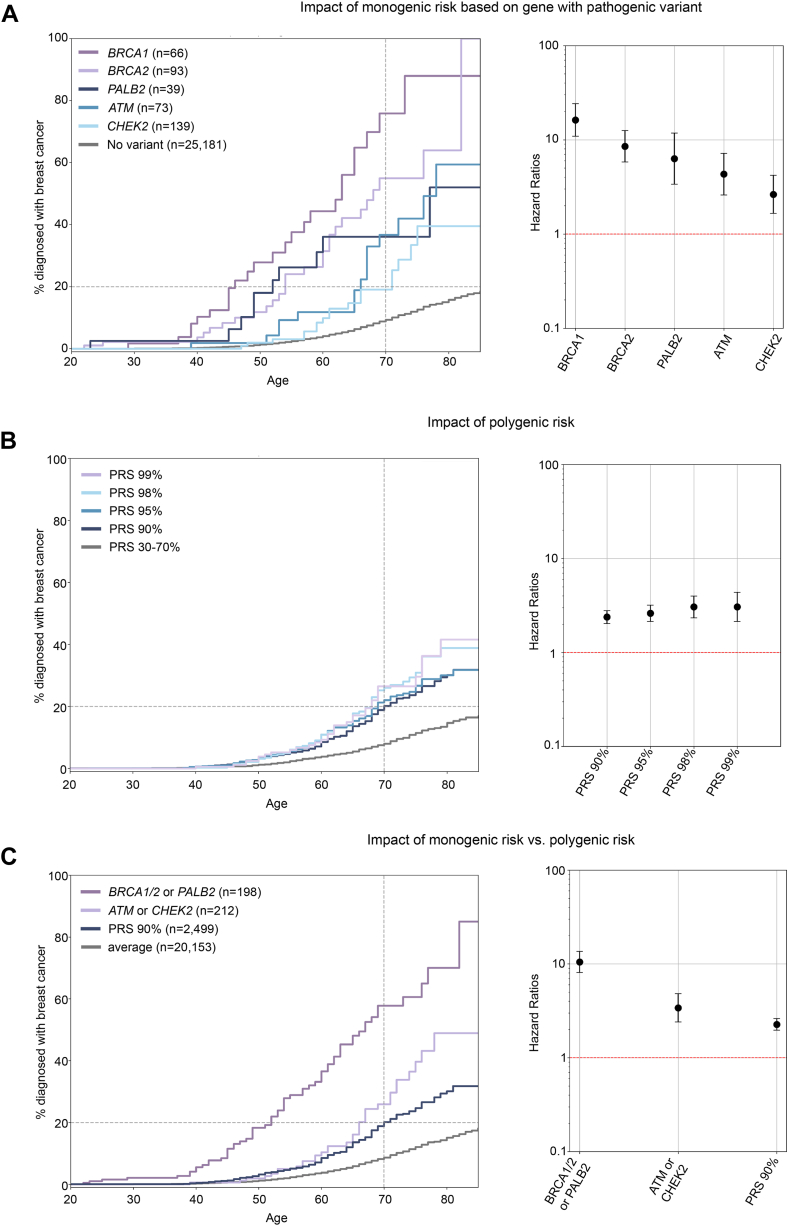
**Impact of monogenic or polygenic risk on breast cancer diagnosis.** A. Left panel: Kaplan Meier curves showing the % of women with a breast cancer diagnosis by age based on whether they have a pathogenic variant in 1 of 5 genes: *BRCA1* (purple curve), *BRCA2* (light purple), *PALB2* (dark blue), *ATM* (blue), or *CHEK2* (light blue). Gray curve: no pathogenic variants. Right panel: Cox proportional hazard ratios and their 95% CI; *y*-axis is on a log scale. B. Left panel: Kaplan Meier curves showing the % of women with a breast cancer diagnosis by age based their breast cancer polygenic risk score: PRS 99% (top 1%) in light purple, PRS 98% (top 2%) in light blue, PRS 95% (top 5%) in blue, PRS 90% (top 10%) in dark blue, PRS 30% to 70% (average score) in gray. Right panel: Cox proportional hazard ratios and their 95% CI; *y*-axis is on a log scale. C. Left panel: Kaplan Meier curves showing the percentage of women with a breast cancer diagnosis by age based on their monogenic or polygenic risk. Purple curve: women with a pathogenic variant in *BRCA1* or *BRCA2* or *PALB2*. Light purple curve: women with a pathogenic variant in *ATM* or *CHEK2*. Dark blue curve: women without a pathogenic variant who are in the top 10% of the PRS distribution. Gray curve: all other women. Right panel: Cox proportional hazard ratios and their 95% CI; *y*-axis is on a log scale.

The risk of breast cancer was highest for women with a pathogenic variant in *BRCA1* or *BRCA2*: hazard ratio (HR) of 16.2 (95% CI: 10.9-24.1, *P* = 2.6e−76) for *BRCA1* and HR = 8.5 (CI: 5.8-12.5, *P* = 1.3e−39) for *BRCA2* (Figure 2A). The next-highest risk was for women having a pathogenic variant in *PALB2*, with a HR of 6.3 (95% CI: 3.4-11.7, *P* = 3.2e−11), followed by *ATM*, with a HR of 4.3 (95% CI: 2.6-7.2, *P* = 1.0e−09), and finally *CHEK2,* with a HR of 2.6 (95% CI: 1.7-4.2, *P* = 2.4e−05) (Figure 2A, Supplemental Table 5). The probability of diagnosis at age 70 was 9.3% for women without a pathogenic variant, 76% for women with a *BRCA1* pathogenic variant, 55% for those with a *BRCA2* pathogenic variant, 36% for those with a *PALB2* pathogenic variant, 37% for those with an *ATM* pathogenic variant, and 19% for those with a *CHEK2* pathogenic variant. This analysis revealed that some individuals with a pathogenic variant in *PALB2* were diagnosed with breast cancer well before the age of 50 (Supplemental Table 5) and had a similar risk to those with a *BRCA2* variant before the age of 50 (Figure 2A) when, until recently, guidelines recommended beginning screening for everyone.

### High polygenic risk significantly increases risk of breast cancer but less so than monogenic risk

Next, we evaluated the clinical impact of having a high polygenic risk for breast cancer, which quantifies the contribution of common variants known to be associated with this disease. We used a validated PRS of 313 SNVs (Supplemental Table 6).^14^ Quality control measures for the SNVs used are depicted in Supplemental Figure S4A and B, and implementation details can be found in the Materials and Methods section. The median breast cancer PRS showed variation based on genetic similarity (Supplemental Table 7). When comparing breast cancer cases and controls, the distributions of the PRS were shifted (Supplemental Figure 4C and D), and the PRS achieved an area under the curve (AUC) of 0.63 in participants in the Europe genetic similarity group and 0.66 in participants in the Americas genetic similarity group, consistent with findings reported in other studies using this PRS.^14^ Although we could not compute the AUC for other genetic similarities due to an insufficient number of participants, subsequent analyses included all participants regardless of their genetic similarity. To enable the study of all participants and to better emulate a real-world scenario in which all participants would receive a result, we assigned a PRS percentile to each participant based on their rank within their genetic similarity group. We then combined all the participants and assessed the impact of being in the top or bottom of their group-specific PRS distribution. Furthermore, we excluded all participants having a pathogenic variant in 1 of the 5 genes previously studied, namely, *BRCA1*, *BRCA2*, *PALB2*, *ATM*, and *CHEK2*, to ensure that these variants would not confound the results.

Women in the top 2% and top 10% of their group-specific PRS distribution exhibited an increased risk of breast cancer compared with women with an average polygenic risk, with HR of 3.1 and 2.4, respectively (CI: 2.3-4.0 and 2.0-2.8, *P* = 6.4e−18 and 1.9e−28, respectively) (Figure 2B, Supplemental Tables 8 and 9). Both of these risks were lower compared with the risk associated with having a pathogenic variant in *BRCA1*, *BRCA2*, or *PALB2* (HR = 10.4, CI: 8.1-13.5) but were closer to the risk associated with having a pathogenic variant in *ATM* or *CHEK2* (HR=3.4, CI: 2.4-4.8) (Figure 2C). For women with a PRS in the top 2% and top 10% of their group-specific distribution and without a pathogenic variant, the probability of diagnosis at age 70 was 26% and 20%, respectively.

### Polygenic risk can modify monogenic risk for genes of intermediate penetrance: *ATM* and *CHEK2*

**Figure 3:**
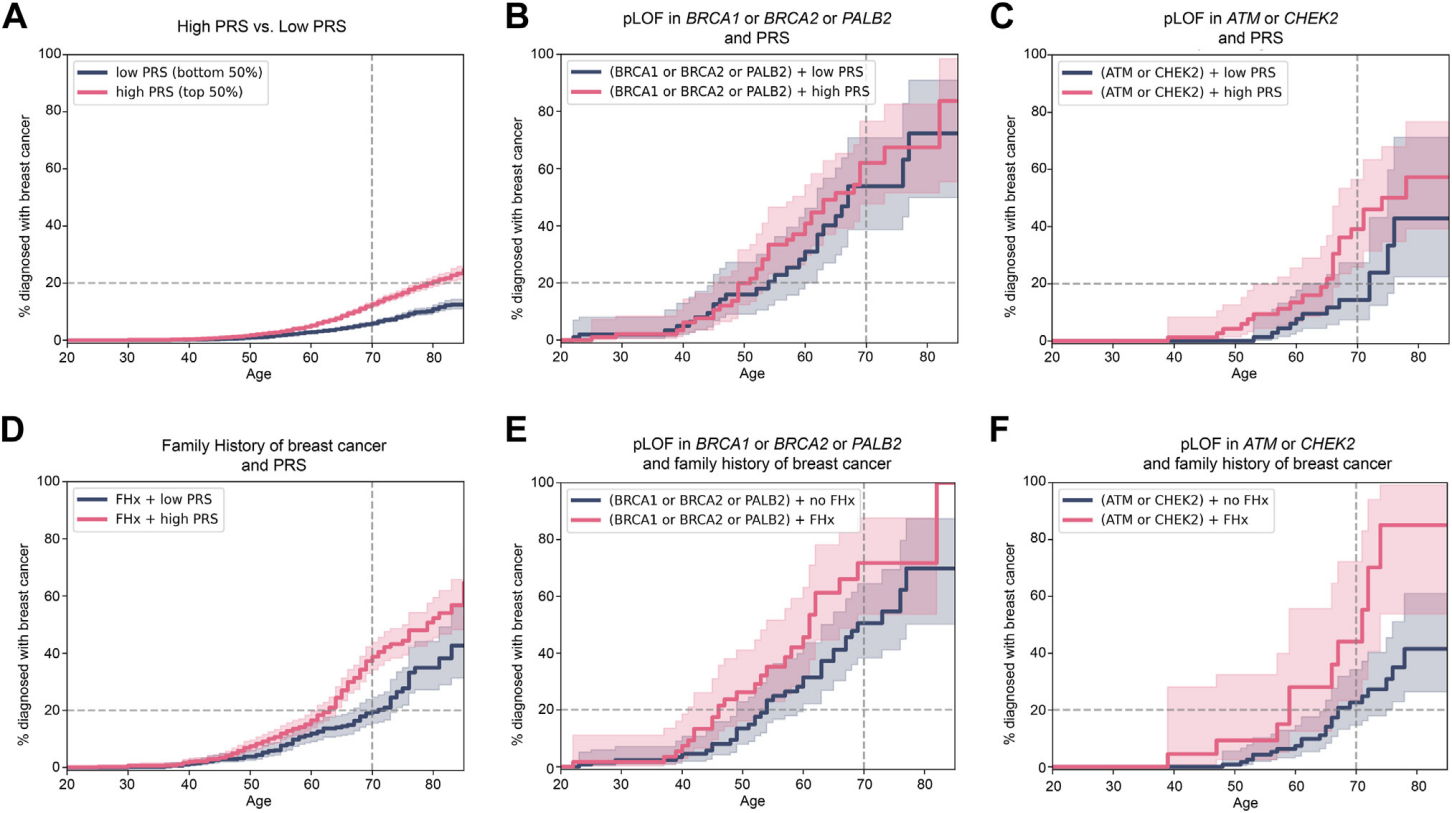
**Impact of combining two non-modifiable risk factors on breast cancer diagnosis.** Kaplan Meier curves showing the percentage of women with a breast cancer diagnosis by age based on (A) being in the top 50% of the PRS (*N =* 12,594; pink curve) vs bottom 50% of the PRS (*N =* 12,587; blue curve). B. Having a pathogenic variant in *BRCA1*, *BRCA2* or *PALB2* and a high PRS (*N =* 100; pink) vs having a pathogenic variant in *BRCA1*, *BRCA2*, or *PALB2* and a low PRS (*N =* 98; blue). C. Having a pathogenic variant in *ATM* or *CHEK2* and a high PRS (*N*= 112; pink) vs having a pathogenic variant in *ATM* or *CHEK2* and a low PRS (*N =* 100; blue). D. Having a family history of breast cancer and a high PRS (*N =* 986; pink) vs having a family history of breast cancer and a low PRS (*N =* 700; blue). E. Having a pathogenic variant in *BRCA1*, *BRCA2*, or *PALB2* and a family history of breast cancer (*N =* 61; pink) vs having a pathogenic variant in *BRCA1*, *BRCA2*, or *PALB2* and no family history of breast cancer (*N =* 137; blue). F. Having a pathogenic variant in *ATM* or *CHEK2* and a family history of breast cancer (*N =* 25; pink) vs having a pathogenic variant in *ATM* or *CHEK2* and no family history of breast cancer (*N =* 187; blue). 95% CI are represented in light pink or blue.

Next, we investigated whether we could enhance the predictive power by combining polygenic risk with other risk factors. To improve statistical power and avoid overly small subgroups, we partitioned the cohort into 2 groups based on PRS. The HR of women in the top 50% of the PRS compared with those in the bottom 50% of the PRS was 2.1 (CI: 1.8-2.3) (Figure 3A, Supplemental Table 10). Although most combinations of polygenic risk with monogenic risk (Figure 3B and C), polygenic risk with family history (Figure 3D), or family history with monogenic risk (Figure 3E and F) enhanced risk stratification, 1 combination—PRS and pLOF in *ATM* or *CHEK2*—appeared particularly useful clinically. This was because individuals in the higher-risk group were clearly above the 20% risk at age 70, a threshold that defines women at high risk,^17^ whereas the lower-risk group was clearly below this threshold. The probability of breast cancer diagnosis at age 70 in individuals with a pathogenic variant in *ATM* of *CHEK2* and in the top 50% of the PRS distribution was 39.2%, whereas those in the bottom 50% of the PRS distribution had a risk of only 14.4% (Figure 3C, Supplemental Table 10). Individuals with a pathogenic variant in *BRCA1*, *BRCA2*, or *PALB2* had a risk of breast cancer well above 20% at age 70, regardless of their PRS (Figure 3B) or family history (Figure 3E, Supplemental Table 10).

Previous studies have demonstrated a correlation between family history and PRS, prompting a reevaluation and adjustment of some weights in the PRS to account for this relationship.^14^^,^^31^ Consequently, we sought to investigate if there were associations between PRS, rare pathogenic variants, and family history in our data. To probe these potential correlations, we excluded individuals with a breast cancer diagnosis and limited the analysis to the group with European genetic similarity to utilize normalized PRS values (instead of percentiles). We discovered a significant association between having a family history of breast cancer and possessing a pathogenic variant (*P* = 3.5e−9, Fisher’s exact test and OR = 3.5). Additionally, we found a positive and significant association between normalized PRS values and having a family history of breast cancer (*P* = 4.6e−09, *t* test), but no association between normalized PRS values and having a pathogenic variant in any of the 5 genes associated with monogenic risk (*P* = 0.74, *t* test). Collectively, these findings suggest that incorporating both PRS and monogenic risk in genes of intermediate penetrance may be advantageous. Conversely, integrating a family history of breast cancer with other risk factors may yield reduced benefits, partially because of the connection between family history of breast cancer with both monogenic risk and PRS.

### Evaluation of different screening strategies based on the combination of family history risk, monogenic risk, and polygenic risk

Although determining the odds ratio for developing breast cancer based on screening results is crucial, implementing genetic screening in the general population necessitates considering additional factors. These include the number of individuals testing positive who will require further counseling and surveillance, the number of individuals overlooked by current guidelines, and those diagnosed with breast cancer before the age of 50 who are missed by genetic screening strategies. Accordingly, we calculated these parameters for various genetic screening strategies and panels within the HNP (Table 2). Based on these figures and current medical practices in the United States, 1 potential strategy (strategy #1) could involve referring all women with either a family history of breast cancer, a pathogenic variant in *BRCA1*, *BRCA2*, and *PALB2*, or a pathogenic variant in *ATM* and *CHEK2* coupled with a high PRS to a high-risk breast cancer clinic (Supplemental Figure 5A, Supplemental Table 11). An alternative approach (strategy #2) could entail discontinuing the ascertainment of family history of breast cancer and instead refer all women with a pathogenic variant in *BRCA1*, *BRCA2*, *PALB2*, *ATM*, and *CHEK2* or a PRS in the top 10% (Supplemental Figure 5B, Supplemental Table 11). The strategy #1 approach had the better PPV of 34%, specificity of 94.3%, and the lower NNS of 183. It is noteworthy that a considerable number of patients diagnosed with breast cancer before the age of 50 would not be identified by any of these screening strategies. Incorporating other independent risk factors such as prior health history, dense breast tissue, and menstrual and reproductive history may aid in identifying some of these patients earlier.^16^^,^^32^^,^^33^

**Table 2:** Impact of different strategies for genetic screening in the entire population

Genetic Strategy Genetic Component or Family History	*N* (% of Population)	Probability of Being Diagnosed With Breast Cancer at Age 70^a^	Number of False Negatives^b^	*N* With a Family History Recorded at Any Time (% of Those Identified by Genetic Strategy)
BRCA1 + BRCA2	159 (0.6%)	63.1%	209	53 (33.3%)
*BRCA1* + *BRCA2* + *PALB2* + (*ATM* + *CHEK2*) × top 50% PRS	298 (1.2%)	51.1%	201	72 (24.2%)
*BRCA1* + *BRCA2* + *PALB2* + *ATM* + *CHEK2*	410 (1.6%)	40.4%	201	86 (21.0%)
*BRCA1* + *BRCA2* + *PALB2* + *ATM* + *CHEK2* + 10% PRS	2,909 (11.4%)	23.2%	156	297 (10.2%)
No genetic test. Family History best case scenario^c^	1,772 (6.9%)	32.9%	139	NA
No genetic test. Family History worse case scenario^d^	1,513 (5.9%)	9.6%	207	NA

a Based on Kaplan Meier curves at age 70.

b False negatives are female participants (out of 25,591) who were diagnosed with breast cancer before age 50 but were not identified by the genetic screening approach.

c If all women with a FHx-BrCa recorded at any time were ascertained before diagnosis.

d Only including women with FHx-BrCa recorded in EHR before a BrCa diagnosis.

## Discussion

The recently updated guidelines from the US Preventive Services Task Force now recommend biennial screening mammography for all women aged 40 to 74 (previously aged 50-74) at average risk. Guidelines suggest that women with a parent, sibling, or child diagnosed with breast cancer, or who have early-onset breast cancer themselves, may benefit from earlier screening. Despite operational and practical challenges, the ascertainment of family history is recommended.^34^^,^^35^ Challenges to ascertaining family history include time constraints during medical visits, smaller family sizes, families with adopted members, families that do not discuss health issues, and male-dominant pedigrees. In a large health system from Nevada, we found a 6.9% prevalence of breast cancer family history. Other studies focusing on family history among women in the general population reported higher prevalence: 16.1% among 222,019 women in the Breast Cancer Surveillance Consortium^36^ and 24.1% based on the National Health Interview Survey.^37^ We also noted an ascertainment bias, as family history of breast cancer often is determined at the time of breast cancer diagnosis. The true risk associated with having a family history of breast cancer is likely lower than the HR of 4.9 we found, once a larger number of individuals with a family history and less biased ascertainment are taken into account. Furthermore, the mean age at the first code for family history of breast cancer was 50.2 years, suggesting a missed opportunity for early-onset breast cancer screening.^38^ Although family history ascertainment can be enhanced with tools such as the FHS-7, which is limited to 7 questions, these tools often have low specificity and might provide an incomplete picture of cancer risk based on family history.^39^^,^^40^ Our findings suggest that risk based on family history is ascertained too late and too infrequently and that more real-world evidence of population genetic screening, which already presents a promising alternative, is needed.^4^^,^^41^

Our results from all-comers population genetic screening confirmed that rare pLOF variants in *BRCA1*, *BRCA2*, *PALB2*, *ATM*, and *CHEK2* confer significant risk to breast cancer in the overall population.^7^^,^^10^^,^^12^^,^^42^^,^^43^ These results provide useful guidance for discussions on breast cancer risk for women with a variant in *ATM* or *CHEK2*, which are less studied. We found that by age 70, women with an *ATM* pLOF variant had a 37% accumulated risk of being diagnosed with breast cancer (HR = 4.3), aligning with the 28% to 38% lifetime risk mentioned in the NCCN guidelines.^18^ Women with a *CHEK2* pLOF had a 19% accumulated risk (HR = 2.6), which is on the lower end of the 23% to 48% lifetime risk mentioned in the NCCN guidelines.^18^ For *ATM* and *CHEK2*, the NCCN guidelines suggest an annual mammogram starting at age 40 but specify insufficient evidence to support risk-reducing mastectomy based on *ATM* or *CHEK2* variant status alone; instead, management should be based on personal risk factors and family history.^18^ Our findings, however, show a significant correlation between family history and having a pathogenic variant, as well as between family history and a high PRS. Adding family history to genetic findings might not substantially increase predictive utility. However, our ability to evaluate this was hampered by the lack of an unbiased baseline family history ascertainment (eg, a large group of women with family history data systematically collected at age 18). On the other hand, we showed that coupling the identification of a variant in *ATM* or *CHEK2* with the calculation of a PRS might effectively identify women most at risk of breast cancer.

To explore polygenic risk in the population, we used a published and validated 313-SNVs PRS for breast cancer^14^ and confirmed this PRS model as a robust candidate for clinical implementation globally.^26^ Individuals within the top decile of polygenic risk exhibit a 20.1% accumulated risk of breast cancer by age 70, corresponding to an approximate 2.5-fold increase in lifetime breast cancer risk. Despite this evidence supporting the value of a PRS as a screening tool, many studies have highlighted challenges associated with their clinical use.^44^^,^^45^ These include (1) the current absence of clinical trials to measure their efficacy and determine the most effective methods for communicating and returning polygenic risk results, (2) the fact that most PRS have been built using data from individuals of Europe genetic similarity and may be less predictive for individuals from other backgrounds, and (3) the larger effect of monogenic risk compared with polygenic risk. Screening for both rare pathogenic variants and polygenic risk and combining these results could address some of these challenges, a use case that has been discussed in other studies and perspectives.^45^, ^46^, ^47^, ^48^ Based on our data from the HNP, a possible screening strategy could focus on women with a pathogenic variant in *BRCA1* or *BRCA2* or *PALB2* and then consider women with a pathogenic variant in genes with slightly lower penetrance (such as *ATM* or *CHEK2*) who also fall within the top 50% of the PRS distribution. This approach identifies women at much higher risk compared with current guidelines and can be integrated with the current state-of-the-art based on family history ascertainment, without substantially increasing the number of individuals identified as high risk.

### Limitations of the study

This study has several limitations. First, the study population is confined to adults residing in Nevada; thus, it may not completely represent the broader US population, let alone global populations. Second, we mostly relied on diagnoses codes in the EHR to obtain family history information. Using additional sources in the EHR would identify more individuals with a family history (Supplemental Figure 2), but it remains difficult to disentangle reasons why family history may be missing or when it is positive, if the extent of family history is extensive enough to warrant a high-risk classification (eg, per NCCN guidelines). Third, our analysis did not capture all individuals with monogenic risk. Specifically, our variant annotation and classification process did not evaluate many rare missense variants, particularly those in *PALB2*, *ATM*, and *CHEK2*. We also did not evaluate variants in additional genes, such as *BARD1*, *CDH1*, *PTEN*, *RAD51C*, *RAD51D*, *STK11*, or *TP53*, which could increase the risk of breast cancer. These genes were not included because (1) there was no statistical significant association between rare coding or rare loss-of-function variants in these genes and breast cancer when looking in the general population^13^^,^^20^ (for some genes such as *TP53*, the lack of association is because of the extreme rarity of loss-of-function variants in the gene) and (2) other studies, such as the WISDOM, have yet to include these genes in their protocol.^49^ Lastly, there are biases in the accuracy of the PRS calculations based on genetic similarity. For example, a study using the same 313-SNVs score found lower AUCs (between 0.53 and 0.57) in women genetically similar to an African population or American population compared with women genetically similar to a European population (AUC of 0.6).^50^ Making sure that PRS have clinical utility for each individual and group of individuals is an important consideration to ensure the clinical utility of this tool for population screening.^47^ These limitations should be considered when interpreting the results of this study and planning for future research to implement new strategies for identifying women at a higher risk of breast cancer.

## Data Availability

The HNP data are available to qualified researchers upon request and with permission of the Institute for Health Innovation (IHI) and Helix. Researchers who would like to obtain the raw genotype data related to this study will be presented with a data user agreement, which requires that no participants will be reidentified and no data will be shared between individuals or uploaded onto public domains. The IHI encourages and collaborates with scientific researchers on an individual basis. Examples of restrictions that will be considered in requests to data access include but are not limited to (1) whether the request comes from an academic institution in good standing and will collaborate with our team to protect the privacy of the participants and the security of the data requested, (2) type and amount of data requested, (3) feasibility of the research suggested, and (4) amount of resource allocation for the IHI and Renown Hospital required to support the collaboration. Any correspondence and data availability requests related to the HNP should be addressed to J.J.G. (jgrzymski@unr.edu).

## ORCIDs

Alexandre Bolze: http://orcid.org/0000-0001-7399-2766

Joseph J. Grzymski: http://orcid.org/0000-0003-2646-8958

## Conflict of Interest

Alexandre Bolze, Kelly M. Schiabor Barrett, Ildiko Thibodeau, Natalie Telis, Ruomu Jiang, Nicole L. Washington, Matthew J. Ferber, Catherine Hajek, and Elizabeth T. Cirulli are employees of Helix. A patent application has been filed by Helix for the “Dynamic risk management for breast cancer based on multi-factor genetic testing” with Alexandre Bolze and Joseph J. Grzymski as inventors, and its current status is unpublished (application number 63/467,250).

## Supplemental Material

There are 5 supplementary figures and 11 supplementary tables.

Table S1 Demographic information for Renown Health patients aged 18-75 who had a clinical encounter with the health system and Healthy Nevada Project participants. Table S2: List of all variants classified as pathogenic. Table S3: List of all copy number variants classified as pathogenic. Table S4: Number of individuals with a pathogenic variant per gene. Table S5: Clinical impact of pathogenic variants on breast cancer diagnosis. Table S6: SNPs and coordinates in GRCh38 for breast cancer polygenic risk score. Table S7: Median breast cancer polygenic risk score for different populations grouped by genetic ancestry. Table S8: Distribution of the polygenic risk score in the Healthy Nevada Project. Table S9: Clinical impact of the polygenic risk score on breast cancer diagnosis. Table S10: Impact of combining two non-modifiable risk factors on breast cancer diagnosis. Table S11: Impact of two potential strategies to identify women at high risk of breast cancer.

Figure S1 Clinical characteristics of the research participants included in this study. Figure S2: Analysis of sources of breast cancer family history records in the EHR. Figure S3: Process for variant annotation, classification and variant call quality control. Figure S4: Implementation of the breast cancer polygenic risk score. Figure S5: Impact of two potential strategies to identify women at high risk of breast cancer in the population.
