# Supplementary material for: Combining rare and common genetic variants improves population risk stratification for breast cancer
Source: Genet Med Open. 2024 Feb 2;2:101826. doi: 10.1016/j.gimo.2024.101826 (PMC11613897; doi:10.1016/j.gimo.2024.101826)
Supplement: Figure S1 — Clinical characteristics of the research participants included in this study. Figure S2: Analysis of sources of breast cancer family history records in the EHR. Figure S3: Process for variant annotation, classification and variant call quality control. Figure S4: Implementation of the breast cancer polygenic risk score. Figure S5: Impact of two potential strategies to identify women at high risk of breast cancer in the population. [file mmc2.pdf]

Supplemental Material

Combining rare and common genetic variants improves population risk stratification for breast cancer

Alexandre Bolze[1], Daniel Kiser[2], Kelly M. Schiabor Barrett[1], Gai Elhanan[2], Jamie M. Schnell Blitstein[3], Iva Neveux[2], Shaun Dabe[3], Harry Reed[2], Alexa Anderson[3], William J. Metcalf[2], Ekaterina Orlova[4], Ildiko Thibodeau[1], Natalie Telis[1], Ruomu Jiang[1], Nicole L. Washington[1], Matthew J. Ferber[1], Catherine Hajek[1], Elizabeth T. Cirulli[1], Joseph J. Grzymski[2,3].

[1]: Helix, San Mateo, CA, USA
[2]: Department of Internal Medicine, University of Nevada Reno, School of Medicine, Reno, NV, USA
[3]: Renown Health, Reno, NV, USA
[4]: Department of Human Genetics, University of Pittsburgh, Pittsburgh, PA, USA

There are 5 supplemental figures and 11 supplemental tables (Tables are in separate Excel file)

Figure S1: Clinical characteristics of the research participants included in this study.

Figure S2: Analysis of sources of breast cancer family history records in the EHR.

Figure S3: Process for variant annotation, classification and variant call quality control.

Figure S4: Implementation of the breast cancer polygenic risk score.

Figure S5: Impact of two potential strategies to identify women at high risk of breast cancer in the population.

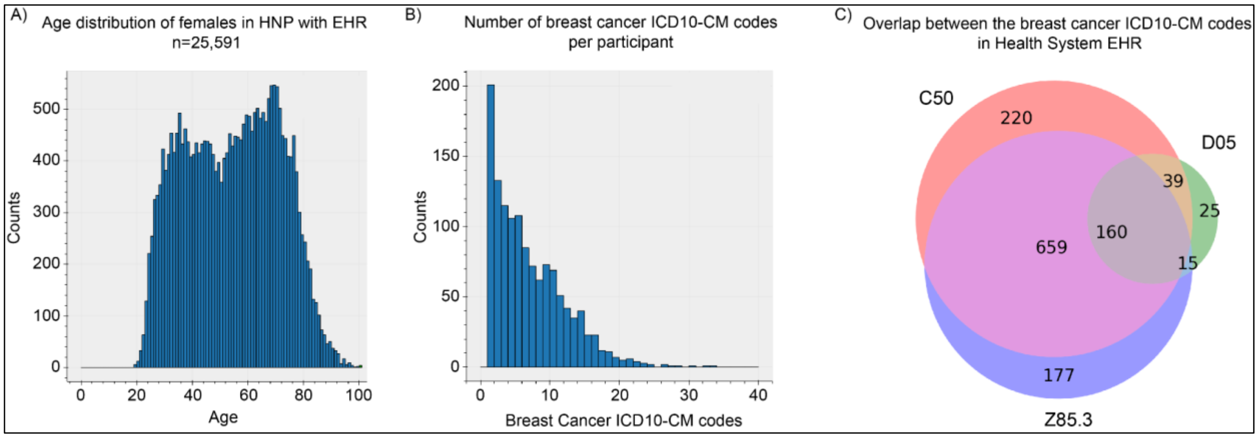

<u>Figure S1:</u> Clinical characteristics of the research participants included in this study. (A) Distribution of age in 2023 in the Healthy Nevada Project female participants. **(B)** Number of entries of ICD10-CM codes indicating a breast cancer diagnosis (ICD10-CM codes starting with C50, D05, or Z85.3) per participant among women with at least 1 of these codes. **(C)** Number of women with at least one of each ICD10-CM code.

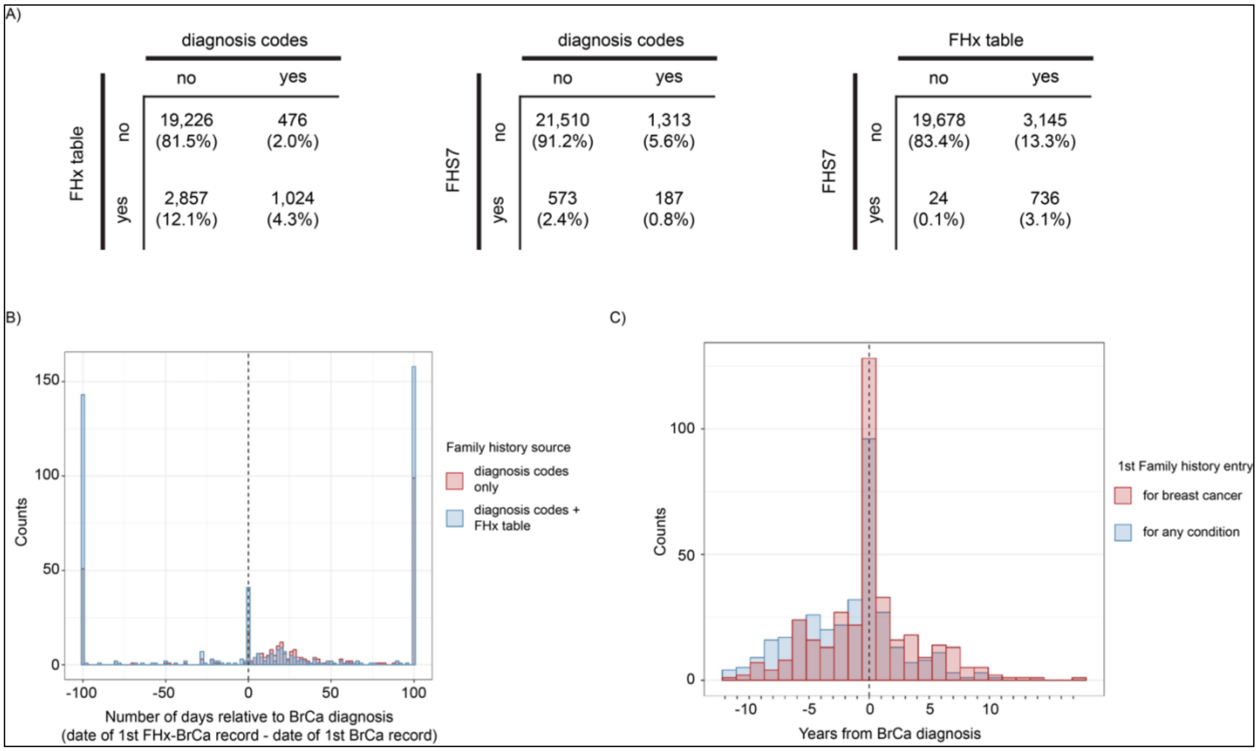

<u>Figure S2:</u> Analysis of sources of breast cancer family history records in the EHR. Analysis done using data from August 2021 for 23,583 Healthy Nevada Project participants. **(A)** Confusion matrices indicating correspondence between data sources documenting a family history of breast cancer ("no" indicating no documentation, "yes" indicating a positive documentation). FHx table is the family history table. FHS7 represents the table containing responses to the seven-question family history screening. **(B)** Temporal distribution of initial documentation of family history for breast cancer relative to date of initial breast cancer diagnosis - a comparison of when only diagnosis codes are used versus when entries from the FHx table are also incorporated. N=316 for diagnosis codes only and N=477 for diagnosis codes + FHx table. **(C)** Temporal distribution of initial FHx table entries for breast cancer versus the temporal distribution of initial FHx table entries for any condition, relative to date of breast cancer diagnosis. N=371, of which 347 (93.5%) also had an family history entry for a condition other than breast cancer.

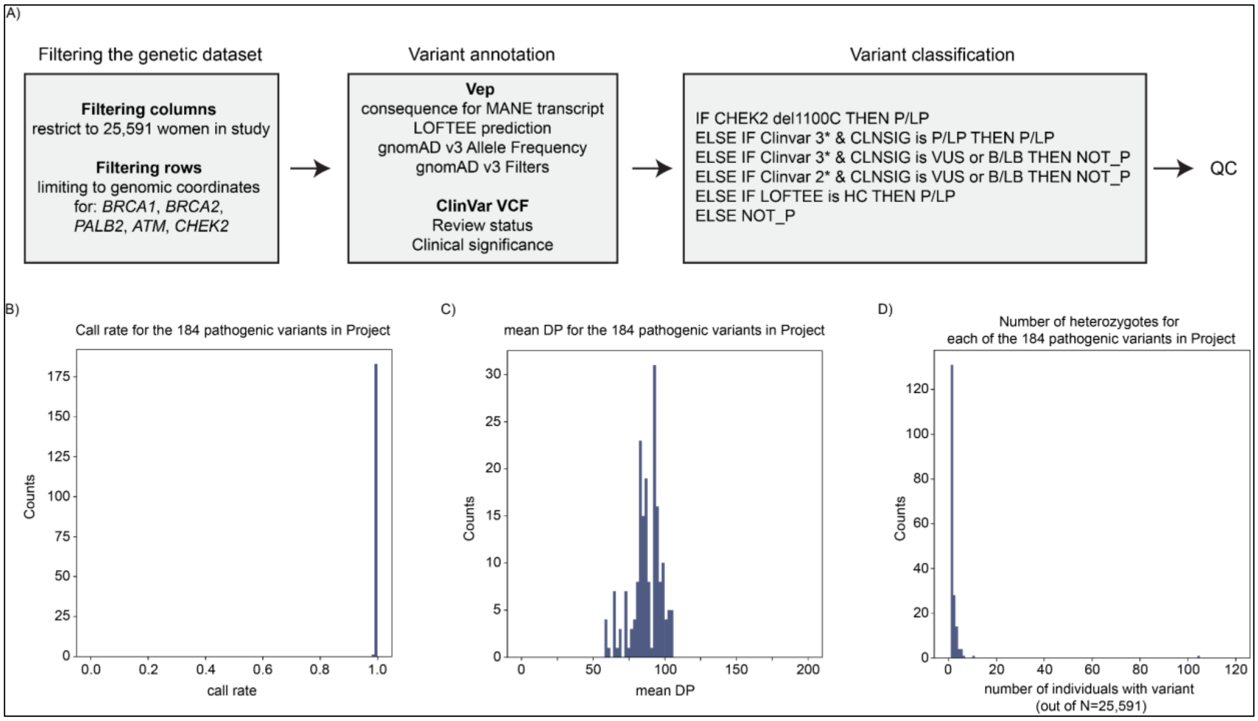

<u>**Figure S3:**</u> **Process for variant annotation, classification and variant call quality control. (A)** Schematic of the workflow for variant annotation and classification. References to some of the tools used are provided in the Material and Methods section. Clinvar 3* represents a Clinvar review status of ‘Reviewed by Clingen expert panel’. Clinvar 2* represents a Clinvar review status of ‘Multiple submitters, no conflicts’. **(B)** Call rate in the study cohort for the 184 variants classified as ‘pathogenic’. **(C)** Mean DP (read depths) for the 184 variants classified as ‘pathogenic’. **(D)** Number of women heterozygotes for each of the 184 variants classified as ‘pathogenic’. The one variant with more than 100 individuals with the variant is *CHEK2* del1100C (ENST00000404276.6:c.1100del).

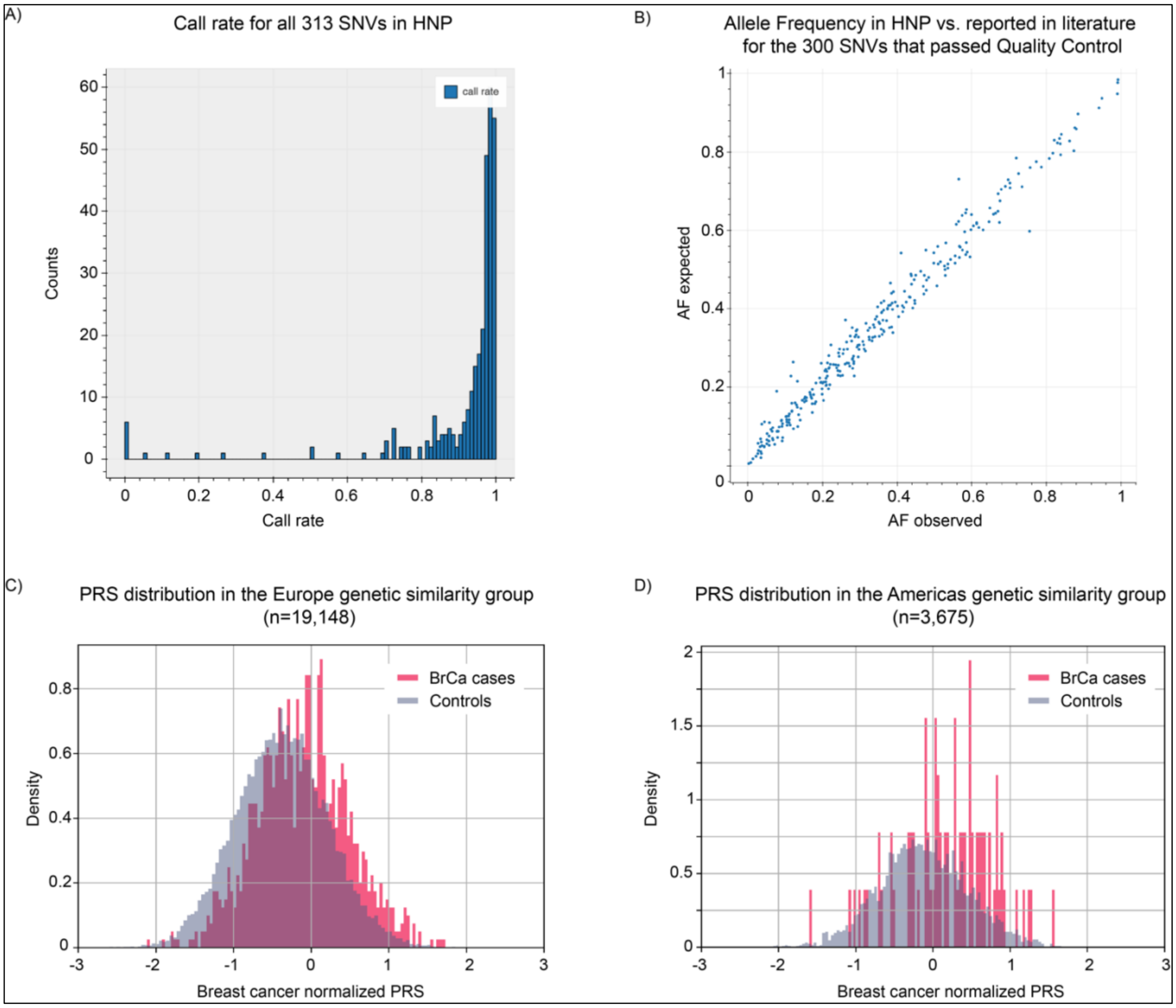

<u>Figure S4:</u> Implementation of the breast cancer polygenic risk score. (A) Call rate in the Healthy Nevada Project for the 313 SNVs included in the PRS model used in this study. Call rate was defined as the fraction of individuals with a high-confidence genotype either derived from direct sequencing or via imputation. **(B)** Scatter plot showing the expected Allele Frequency (AF) compared to the observed AF in the Healthy Nevada Project participants. The expected AF comes from the original paper that published this score[1]. Each dot is one of the 300 SNVs that passed our internal quality control. **(C and D)** Distribution of the normalized PRS values in breast cancer cases (pink) and in controls (blue). Panel C represents individuals with Europe genetic similarity. Panel D represents individuals with Americas genetic similarity.

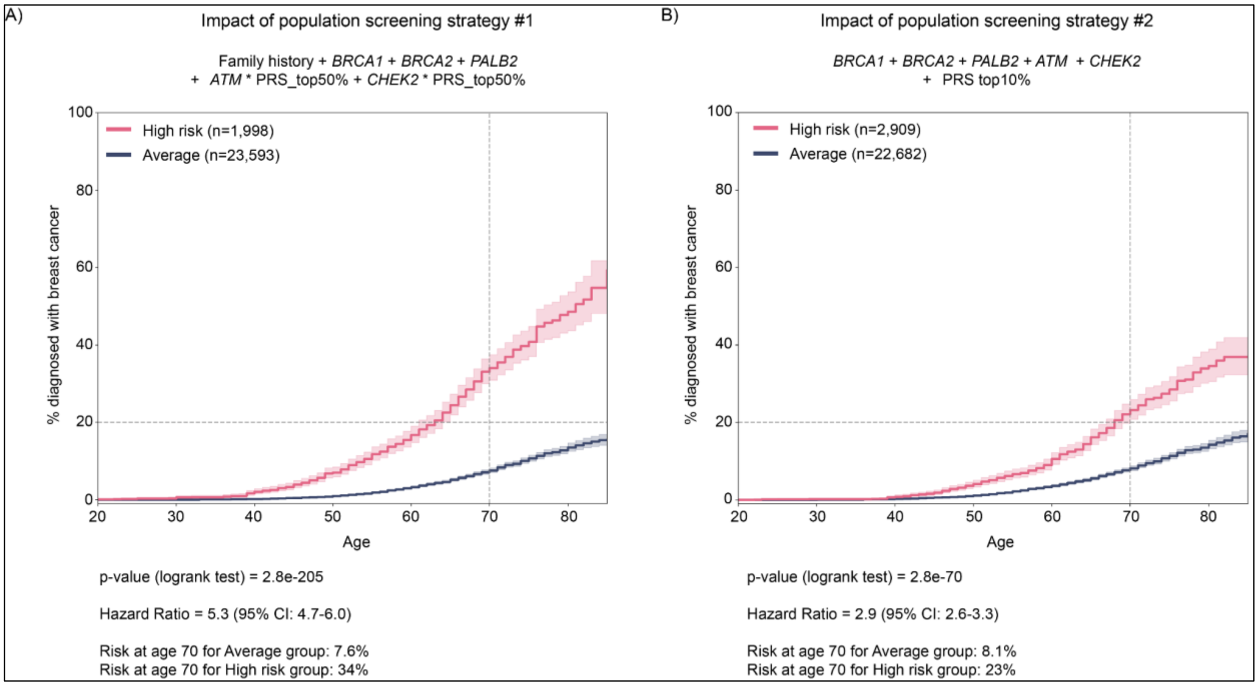

Figure S5: Impact of two potential strategies to identify women at high risk of breast cancer in the population. **A)** Kaplan Meier curves showing the % of women with a breast cancer diagnosis by age based on whether they were identified as 'high risk' (pink curve) or at average risk (blue curve) with strategy #1. **(B)** Kaplan Meier curves showing the % of women with a breast cancer diagnosis by age based on whether they were identified as 'high risk' (pink curve) or at average risk (blue curve) with strategy #2.
